# Complexity and clinical significance of drug–drug interactions (DDIs) in oncology: challenging issues in the care of patients regarding cancer-associated thrombosis (CAT)

**DOI:** 10.1007/s00520-022-07235-8

**Published:** 2022-08-06

**Authors:** Nikolaos Tsoukalas, Norman Brito-Dellan, Carme Font, Taylor Butler, Cristhiam M. Rojas-Hernandez, Thomas Butler, Carmen Escalante, on behalf of the MASCC Hemostasis Study Group

**Affiliations:** 1 Medical Oncology Department, 401 General Military Hospital of Athens, Athens, Greece; 2 Henry Dunant Hospital Center, Athens, Greece; 3 Hospital Medicine Department, The University of Texas M.D. Anderson Cancer Center, TX Houston, USA; 4 Medical Oncology Department, Hospital Clinic Barcelona, Barcelona, Spain; 5 Vanderbilt University Medical Center, Nashville, TN USA; 6 Section of Benign Hematology, The University of Texas M.D. Anderson Cancer Center, TX Houston, USA; 7 University of South Alabama Mitchell Cancer Institute, Mobile, Alabama USA; 8 General Internal Medicine Department, The University of Texas M.D. Anderson Cancer Center, TX Houston, USA

**Keywords:** Drug-Drug Interactions (DDIs), Cancer-Associated Thrombosis (CAT), Oncology, Anticoagulant, Bleeding, Low Molecular Weight Heparins (LMWHs), Direct Oral Anticoagulants (DOACs)

## Abstract

Cancer patients have an increased risk of developing venous thromboembolic events. Anticoagulation management includes prophylactic or therapeutic doses of low molecular weight heparins (LMWHs) or direct oral anticoagulants (DOACs). However, the management of thrombosis in patients with cancer is complex due to various individual and disease-related factors, including drug–drug interactions (DDIs). Furthermore, DDIs may impact both, cancer and venous thrombosis, treatment effectiveness and safety; their relevance is highlighted by the advances in cancer therapeutics. Given that these new oncology drugs are extensively used, more attention should be given to monitoring potential DDIs to minimize risks. Recognition of DDIs is of utmost importance in an era of rapid developments in cancer treatments and introduction of novel treatments and protocols. When managing cancer-associated thrombosis (CAT), the concomitant use of a DOAC and a moderate or strong modulator (inhibitor or inducer) of CYP3A4 or a P-glycoprotein (P-gp) is most likely to be associated with significant DDIs. Therefore, LMWHs remain the first-line option for the long-term management of CAT under these circumstances and physicians must consider utilizing LMWHs as first line. This review describes the risk of DDIs and their potential impact and outcomes in patients with cancer associated thrombosis (CAT) receiving anticoagulation.

## Introduction

Patients with active cancer have a four- to sevenfold increased risk of experiencing venous thromboembolism (VTE), which includes both deep-vein thrombosis (DVT) and pulmonary embolism (PE) [1, 2]. Anticoagulation is the cornerstone of VTE management regardless of cancer status. Various professional guidelines recommend the use of either low molecular weight heparins (LMWHs, e.g., dalteparin, enoxaparin, tinzaparin, bemiparin) or oral anticoagulants including vitamin K antagonists (VKA) and direct oral anticoagulants (DOACs, e.g., apixaban, edoxaban, rivaroxaban) [3–5]. However, potential drug–drug interactions (DDIs) should be considered before initiating anticoagulation and reviewed periodically during follow-up.

The purpose of this narrative review is to describe the main DDIs mechanisms that challenge clinicians for the proper management of the patient regarding cancer associated thrombosis (CAT). We summarize the most relevant evidence currently available in this topic, focusing on practical daily care situations in oncology.

## General Mechanisms of Potential Drug–Drug Interactions (DDIs)

There are several types of DDIs: pharmacodynamic DDIs, pharmacokinetic DDIs, drug transporters DDIs, human plasma protein DDIs, and absorption DDIs (Table 1).

- Pharmacodynamic interactions may be the ones with the most severe repercussions. These DDIs are the product of the additive effects of the two associated drugs, resulting in therapeutic effects or toxicities when mixed with other drugs or due to an additive effect with mutual potentiation of toxic effects.
- There are several types of pharmacokinetic interactions. The main type is involving drug metabolism by the cytochrome P450 (CYP) enzyme system. Other types are pharmacokinetic interaction concern drug transporters, human plasma protein and absorption.
  Metabolic pharmacokinetic interactions are primarily linked with drug metabolism, especially in the liver. This system has several enzymes, including CYP3A4, which is often involved in drug metabolism. Other CYP enzymes can also interact, such as CYP2D6, 1A2, or 2C8. The pharmacokinetic DDI between two drugs may involve different mechanisms: 1) Drugs A and B are metabolized by the same CYP and thus compete, resulting in a reduction or slowdown of the metabolism of A, B, or both. 2) Drug A inhibits the metabolism of drug B, leading to the risk of an overdose by drug B because of the reduction of its metabolism. 3) Drug A induces the metabolism of drug B, potentially causing subtherapeutic levels of drug B because of the acceleration of its metabolism. It is also essential to remember that the cytochrome P450 enzymes are present in most body tissues, including the intestines. Consequently, there is also a risk of DDI in the intestinal compartment [6].
- Drug transporter-related DDIs are also common. If two drugs are substrates of the same transporter, competition may result in a reduced transport of one drug, or both drugs. Furthermore, the co-administration of an inhibitor of the transport with a substrate of the same transport may lead to increase of the substrate blood level, leading to potential adverse events. Finally, the co-administration of an inducer of the transport with a substrate of the same transport may lead to decrease of the substrate blood level, leading to potential inefficacy. During the metabolization phase, drug transporters like P-gp and BCRP play a significant role. Other drug efflux transporters that may influence bioavailability drugs are the multi-drug resistance protein subfamily (ATP-binding cassette subfamily C member 1 to 12, ABCC1 to 12, like MRP1) and the multi-antimicrobial extrusion protein (MATE), while several uptake transporters may be involved as well [e.g., organic anion transporting peptides (OATPs), organic anion transporters (OATs), and organic cation transporters (OCTs)] [7]. It is also important to mention that inhibitors/inducers of CYP isoenzymes are more and more descripted as weak, moderate and strong. However, it is not always clear to understand the meaning of this classification. According to the EMA, enzyme inhibitors may be classified based on their potency. A strong inhibitor causes a > fivefold increase in the plasma AUC values or ≥ 80% decrease in oral clearance, a moderate inhibitor causes a > twofold increase in the plasma AUC or 50% - ≤ inhibition of oral clearance, and a mild inhibitor causes 1.25 to twofold increase in the plasma AUC or ≤ 50% inhibition of oral clearance.
- Human plasma protein-related DDIs such as plasmatic albumin and alpha-1 acid glycoprotein also involve drug transport across cell membranes [8, 9]. Diminished plasma drug binding by the co-administered drugs is usually a result of either competitive displacement from the same binding site or allosteric displacement following microenvironmental changes at the binding site. Finally, drug binding to albumin is decreased, especially in renal and liver diseases and may be affected by the nutritional status of the patient.
- Absorption-related DDIs can lead to a change in drug bioavailability. Many factors are involved in intestinal absorption, like gastric pH and intestinal environments. Many weak base drugs exhibit pH-dependent solubility. Generally, the solubility of a weak base drug decreases as the pH increases. Thus, when weak base drugs are administered orally, an elevation of the gastric pH induced by another drug or disease state may decrease the weak base drug's absorption, resulting in reduced systemic exposure to the drug [10].

**Table 1:** Types of drug–drug interactions (DDIs)

Type of DDIs	Main proteins involved	Mechanisms
Pharmacodynamic interactions	-	Additive effects of the two drugs
Drug transporter DDIs	BCRP, OATP, OCT, MATE, OATP, P-gp	Inhibition of the transporter leads to a higher drug blood concentration Induction of the transporter leads to a lower drug blood concentration
Pharmacokinetic interactions	CYP3A4 (mainly), CYP2C9,…	Inhibition of the CYP3A4 leads to a higher drug blood concentration Induction of the CYP3A4 leads to a lower drug blood concentration
Human plasma protein DDIs	Albumin, alpha 1-glycoprotein	Competition on the binding site of a protein carrier change the drug concentration because only the free drug is active
Absorption DDIs	-	Intragastric pH influences the solubility and bioavailability of drugs

BCRP: breast cancer resistance protein; DDIs: drug–drug interactions; MATE: multi-antimicrobial extrusion protein; OAT: organic anion transporters; OCT: organic cation transporters; P-gp: P-glycoprotein

## Pharmacodynamic DDIs

Pharmacodynamic DDIs are linked to all anticoagulants, and they are not so easy to anticipate even though they are common.

One study included patients with advanced cancer treated with concurrent VEGFR TKIs and factor Xa inhibitors [11]. Perturbation of the tumor-associated endothelial cells (and potentially non-cancerous tissue) and disturbance of platelet function by VEGFR TKIs may modulate the activation of the coagulation cascade, leading to both bleeding and thromboembolic risks. The authors reported that among 86 cancer patients mainly treated with LMWHs, DOACs, or LMWHs plus DOACs, there was a higher overall bleeding rate in patients anticoagulated and concomitant use of VEGFR-TKIs compared to patients who were receiving only anticoagulants (HR 2.45 [1.28-1.69]). A sub-analysis demonstrated that this higher rate of bleeding was not significant when comparing LMWHs *vs.* LMWHs plus VEGFR TKI (HR 1.85 [0.90-3.81]) and when investigating DOACs vs. DOACs plus VEGFR TKI (HR 4.16 [0.38-45.00]). Besides the fact that the HR was higher with DOACs, suggesting a possible higher risk of bleeding with DOACs for the entire follow-up period (around 30 months), the 6-months analysis reported a higher significant risk of bleeding in the LMWHs group.

## Pharmacokinetic DDIs and Drug Transporter DDIs 

One crucial difference between LMWHs and DOACs is their metabolism and pharmacokinetic parameters (Table 2) [12]. Indeed, the metabolism of DOACs includes CYP3A4 and P-glycoprotein (P-gp). However, it is not the case for LMWHs as their metabolism does not involve CYP3A4 and P-gp [12].

**Table 2:** Main pharmacokinetic characteristics of anticoagulants [12]

Anticoagulant	Target	Protein Binding	Metabolism	Efflux Protein	Inducer/inhibitor of CYP/P-gp
LMWH	AntiXa/AntiIIa	-	Desulfation and depolymerisation	-	No
Rivaroxaban	AntiXa	95%	CYP3A4/3A5/2J2	P-gp, BRCP	No
Apixaban	AntiXa	87%	CYP3A4/3A5	P-gp, BRCP	No
Edoxaban	AntiXa	42-59%	CYP3A4	P-gp, BRCP	No

Consequently, a potentially important safety consideration in using any DOACs in CAT is the risk for DDIs with systemic anticancer treatments, including chemotherapeutic agents, hormonal therapy, immunotherapy, and drugs used in supportive care like dexamethasone or antiemetic drugs. Potent inhibitors or inducers of P-gp and potent inhibitors or inducers of cytochrome CYP3A4 can interact with DOACs [5, 12] and many anticancer drugs are substrate, inhibitor, and/or inducer of the CYP3A4 and P-gp [13]. Additional clinically significant DDIs data may emerge over time [14].

It must be taken into account that drugs with strong DDIs potential were excluded in DOACs trials for CAT, including drugs commonly used in cancer treatment and supportive care. Regular clinical practice requires understanding and careful assessment of patients who are exposed to these drugs. The clinical impact of such DDIs with strong CYP3A4/P-gp inhibitors was described in a retrospective study concluded that among patients receiving DOACs, concurrent use of clarithromycin was associated with a significantly greater risk of hospital admission with significant bleeding [15].

It is also essential to consider that both moderate inhibitors of CYP3A4/P-gp and not only the strong ones can impact the bleeding rate in VTE patients. Hanigan S et al. reported that DDIs led to significantly more bleeding in an atrial fibrillation (AFib) population treated with apixaban or rivaroxaban. The same publication reported that the increased risk of bleeding started shortly after the exposure to a DDI and increased over time [16]. Another study [17] reported that apixaban in AFib patients exposed to CYP3A4 and/or P-gp inhibitors also had an increased risk for severe bleeding (HR 1.23; 1.01–1.5), whereas a significant effect for patients treated with rivaroxaban or dabigatran and these interacting drugs could not be established (rivaroxaban, HR 1.24; 0.94–1.65, and dabigatran HR 0.84; 0.48–1.45).

Interestingly, the TacDOAC registry [18] reported the bleeding rates among 202 cancer patients treated with DOACs (mainly apixaban and rivaroxaban) and receiving anticancer targeted therapies (Tables 3 and 4). Bleeding rates were analyzed according to the class of anticancer drugs. Several of the anticancer drugs were weak or moderate inhibitors of CYP3A4 with or without P-gp inhibition properties. Major bleeding (MB) rates ranged from 2.3% to 9.5%, and clinically relevant non-major bleeding (CRNMB) rates ranged from 2.3% to 14.3%. These data should be interpreted with caution, as there was no control group. However, when looking at the SELECT-D (rivaroxaban) and CARAVAGGIO/ADAM VTE (apixaban) trials, the TacDOAC bleeding rates seemed higher than the bleeding rates of those 3 RCTs. The authors concluded that concomitant use of different targeted anticancer therapies with DOACs resulted in different bleeding risks, with Bruton's tyrosine kinase (BTK) inhibitors and vascular endothelial growth factor (VEGF) inhibitors found to be associated with higher risks.

**Table 3:** Potential drug–drug interactions of anticancer drugs [21, 22, 28, 42, 49–51]

Cancer type	Anticancer drugs	CYP3A4 interactions	P-gp interactions	BCRP interaction	Protein binding
Anal, Biliary, Head&Neck, Breast, Cervical, Colorectal, Oesophagus, Gastric, NETs, Pancreas	5-FU	No	No	No	No
Prostate	Abiraterone	Moderate inhibitor	Strong inhibitor	No	99.8%
Lymphoma	Acalabrutinib	Weak inhibitor	Substrate	Substrate	97.5%
Ewing, Osteosarcoma, Sarcomas	Actinomycin-D	No	No	No	No
Lung	Afatinib	No	Inhibitor	Substrate and inhibitor	95%
Colorectal	Aflibercept	No	No	No	No
Lung	Alectinib	No	Inhibitor and substrate (M4)	Inhibitor	No
Breast	Anastrozole	Weak/moderate inhibitor	No	No	Not highly
Bladder, Lung	Atezolizumab	No	No	No	No
Bladder	Avelumab	No	No	No	No
Kidney	Axitinib	Substrate	No	Substrate	>99%
Brain, Breast, Cervical, Colorectal, Kidney, Lung, Mesothelioma, Ovarian	Bevacizumab	No	No	No	No
Melanoma	Binimetinib	No	Substrate	Substrate	97.2%
Testicular, Osteosarcoma	Bleomycin	No	No	No	Limited extend
Lung	Brigatinib	Inducer and substrate	Substrate	Substrate	91%
Prostate	Cabazitaxel	No	Inhibitor	Inhibitor	89-92%
Kidney, Thyroid	Cabozantinib	Weak inhibitor and substrate	Inhibitor and substrate	Inhibitor	99.7%
Anal, Cervical, Colorectal, CUP, Biliary, Breast, Oesophagus, Gastric, Pancreas, Thymoma	Capecitabine	No	No	No	10-62%
Biliary, Bladder, Breast, Cervical, CUP, Oesophagus, Gastric, Head&Neck, Lung, Melanoma, Mesothelioma, Osteosarcoma, Ovarian, Pancreas, Testicular, Thymoma, Uterus, Prostate	Carboplatin	No	No	No	29-90%
Brain	Carmustine	No	No	No	No
Lung	Ceritinib	Weak/strong inhibitor	Substrate	Inhibitor	97%
Colorectal, Head&Neck	Cetuximab	No	No	No	No
Anal, Biliary, Bladder, Breast, Cervical, CUP, Oesophagous, Gastric Head&Neck, Liver, Lung, Melanoma, Mesothelioma, NETs, Osteosarcoma, Ovarian, Pancreas, Prostate, Testicular, Thymoma, Thyroid, Uterus	Cisplatin	No	No	No	90%
Melanoma	Cobimetinib	Subtract	Substrate	Inhibitor	94.8%
Lung	Crizotinib	Moderate inhibitor and substrate	Strong inhibitor and substrate	No	91%
Bladder, Breast, Cervical, Ewing, Head&Neck, Lung, Sarcomas, Thymoma, Osteosarcoma, Ovarian	Cyclophosphamide	Weak inhibitor/inducer and substrate	No	No	20-70%
Lung, Melanoma	Dabrafenib	Moderate inducer/transient inhibitor and substrate	Substrate	Inhibitor and substrate	99.7%
Melanoma, Sarcomas	Dacarbazine	No	No	No	5%
Biliary, Bladder, Breast, CUP, Head&Neck, Pancreas, Oesophagus, Gastric, Lung, Ovarian, Prostate, Sarcomas	Docetaxel	Weak inhibitor/weak inducer and substrate	No	No	95%
Bladder, Breast, Cervical, Ewing, Head&Neck, Lung, Liver NETs, Osteosarcoma, Ovarian, Sarcomas, Thymoma, Thyroid, Uterus	Doxorubicin	Weak inhibitor and substrate	Inducer / strong inhibitor and substrate	No	70%
Lung	Durvalumab	No	No	No	No
Colorectal, Melanoma	Encorafenib	Inhibitor/inducer and substrate	Inhibitor and substrate	Inhibitor	86.1%
Prostate	Enzalutamide	Strong inducer and substrate	Strong inducer	Substrate (M5)	>99%
Bladder, Breast, Cervical, Oesophagus, Gastric, Lung, Ovarian, Sarcomas, Uterus	Epirubicin	No	No	No	80%
Breast, Sarcomas	Eribulin	No	No	No	49-65%
Lung, Pancreas	Erlotinib	Inhibitor and substrate	Inhibitor and substrate	Substrate	95%
Prostate	Estramustin	No	Inhibitor	No	No
CUP, Ewing, Lung, NETs, Osteosarcoma, Ovarian, Testicular, Thymoma	Etoposide	Inducer/weak inhibitor	Inhibitor	No	97%
Breast, Kidney, NETs	Everolimus	Weak inhibitor and substrate	Inhibitor and substrate	No	74%
Breast	Exemestane	Substrate	No	No	90%
Melanoma	Fotemustin	No	No	No	25-30%
Breast	Fulvestrant	Substrate	No	No	99%
Lung	Gefitinib	Inhibitor and substrate	Inhibitor and substrate	Inhibitor and substrate	90%
Biliary, Bladder, Breast, Cervical, CUP, Kidney, Liver, Lung, Ovarian Pancreas, Sarcomas, Testicular, Thymoma, Uterus	Gemcitabine	No	No	No	No
Head&Neck	Hydroxyourea	No	No	No	No
Lung	Ibrutinib	Weak inhibitor and substrate	Inhibitor and substrate	Inhibitor	97.3%
Cervical, Ewing, Head&Neck, Kidney, Lung, Osteosarcoma, Sarcomas, Testicular, Thymoma, Uterus	Ifosfamide	Weak inhibitor and substrate	No	No	N/A
GIST	Imatinib	Moderate inhibitor and substrate	Strong inhibitor and substrate	Inhibitor and substrate	95%
Kidney, Liver, Melanoma, NETs	Interferons	No	No	No	No
Melanoma	Ipilimumab	No	No	N/A	N/A
Brain, Colorectal, CUP, Oesophagus, Gastric, Ovarian, Pancreas, Testicular, Thymoma	Irinotecan	Substrate	Substrate	No	65-95%
NETs	Lanreotide	Inhibitor	No	No	No
Breast	Lapatinib	Weak-mild inhibitor and substrate	Moderate-strong inhibitor and substrate	Inhibitor and substrate	>99%
Kidney, Liver, Thyroid	Lenvatinib	Weak inducer/weak inhibitor	Substrate	Substrate	98-99%
Breast	Letrozole	Substrate	No	No	60%
Brain	Lomustine	Mild inhibitor	No	No	No
Breast, Uterus	Megestrol acetate	No	No	No	No
Ewing, Osteosarcoma, Sarcomas, Testicular	Mesna	No	No	No	No
Bladder, Breast, Head&Neck, Osteosarcoma, Testicular	Methotrexate	No	Substrate	Substrate	50%
Osteosarcoma	Mifamurtide	No	No	No	No
Anal	Mitomycin	No	Substrate	No	No
Prostate	Mitoxantrone	No	No	Substrate	78%
Lung	Mobocertinib	N/A	N/A	N/A	N/A
Breast, Lung, Pancreas	Nab-paclitaxel	Moderate inducer and substrate	Inhibitor and substrate	No	No
Breast	Neratinib	Substrate	Substrate and inhibitor	Inhibitor	98-99%
CML	Nilotinib	Moderate inhibitor and substrate	Inhibitor and substrate	Inhibitor and substrate	98%
Head&Neck, Lung	Nintedanib	No	Strong inhibitor and substrate	No	97.8%
Bladder, Colorectal, Gastric, Head&Neck, Liver, Lung, Oesophagus, Melanoma	Nivolumab	No	No	No	No
Thymoma, NETs	Octreotide	No	No	No	65%
Breast, Ovarian, Prostate	Olaparib	Weak inhibitor and substrate	No	Inhibitor	70-91%
Sarcomas	Olaratumab	No	No	No	No
Lung	Osimertinib	Weak inhibitor and substrate	Inhibitor and substrate	Inhibitor	94.7%
Anal, Biliary, Colorectal, CUP, Oesophagus, Gastric, Liver, Ovarian, Pancreas, Testicular	Oxaliplatin	No	No	No	No
Biliary, Bladder, Breast, Cervical, CUP, Gastric, Head&Neck,Lung, Melanoma, Oesophagus, Ovarian, Sarcomas, Testicular, Thyroid, Thymoma, Uterus	Paclitaxel	Moderate inducer and substrate	Inhibitor and substrate	No	94%
Breast	Palbociclib	Weak inhibitor and substrate	Inhibitor and substrate	Inhibitor	85%
Colorectal	Panitumumab	No	No	No	No
Kidney, Sarcomas	Pazopanib	Weak inhibitor	Moderate inhibitor substrate	Substrate	99%
Bladder, Breast, Colorectal, Gastric, Head&Neck, Liver, Lung, Melanoma, Mesothelioma, Oesophagus	Pembrolizumab	No	No	No	No
Lung, Mesothelioma	Pemetrexed	No	No	No	81%
Breast	Pertuzumab	No	No	No	No
Brain	Procarbazine	No	No	No	No
Prostate	Radium 223	No	No	No	No
Mesothelioma	Raltitrexed	No	No	No	93%
Colorectal, Gastric	Ramucirumab	No	No	No	No
Colorectal, GIST, Liver	Regorafenib	Inhibitor and substrate	Inhibitor and substrate	Inhibitor	>99%
Ovarian	Rucaparib	Weak inhibitor and substrate	Inhibitor and substrate	Substrate and weak inhibitor	70.2%
Gastric	S-1 (Tegafur, gimeracil, oteracil)	No	No	No	52% (Tegafur)
Skin	Sonidegib	Substrate	No	Inhibitor	>97%
GIST, Kidney, Liver, Melanoma,Thyroid	Sorafenib	Inhibitor and substrate	Inhibitor and substrate	No	99%
NETs	Streptozocin	N/A	N/A	N/A	N/A
GIST, Kidney, NETs, Thymoma, Thyroid	Sunitinib	Inhibitor and substrate	Inhibitor and substrate	No	90-95%
Breast, Ovarian	Tamoxifen	Mild inhibitor/inducer and substrate	Strong inhibitor / inducer	No	>99%
Brain, Melanoma	Temozolomide	Mild inhibitor	Inhibitor	No	10-20%
Kidney	Temsirolimus	Weak-mild inhibitor and substrate	Inhibitor and substrate	No	40-85%
Cervical, Lung, Ovarian	Topotecan	Inhibitor/inducer	Inhibitor	No	35%
Ovarian, Sarcomas	Trabectedin	Substrate	Substrate	No	94-97%
Lung, Melanoma	Trametinib	Inducer	Inhibitor and substrate	Transient inhibitor	97.4%
Breast, Gastric	Trastuzumab	No	No	No	No
Breast	Trastuzumab emtansine	Substrate	Substrate	No	No
Mesothelioma	Tremelimumab	No	No	No	No
Colorectal	Trifluridine/Tipiracil	No	No	No	96%/8%
Colorectal	UFT (Tegafur/uracile)	No	No	No	52%
Thyroid	Vandetanib	Substrate	Strong inhibitor	Inhibitor	90-94%
Lung, Melanoma	Vemurafenib	Moderate inducer and substrate	Inhibitor and substrate	Inhibitor and substrate	99%
Bladder, Melanoma, Testicular	Vinblastine	Weak inhibitor/inducer and substrate	Strong inducer and substrate	No	No
Brain, Ewing, Lung, Sarcomas, Thymoma	Vincristine	Mild inducer/inhibitor and substrate	Inducer/inhibitor and substrate	No	75%
Melanoma	Vindesine	Substrate	No	Inhibitor	No
Bladder	Vinflunine	Substrate	No	No	67.2%
Breast, Cervical, Lung, Ovarian Prostate, Sarcomas Mesothelioma	Vinorelbine	Weak inhibitor and substrate	No	No	13.5%
Basal cell carcinoma	Vismodegib	No	No	Inhibitor	97%

CUP: cancer of unknown primary; NET: neuroendocrine tumor

Summaries of product characteristic were also used. There is some conflicting statement among the publications. When the information regarding the strength of inhibition/induction was available, it was reported in the table.

**Table 4:** Potential drug–drug interactions of supportive oncology care drugs [22, 23, 29, 43, 50–52]

Classes	Drugs	CYP3A4 interactions	P-gp interactions	BCRP interaction	Protein binding
Corticoid	Dexamethasone	Strong inducer and substrate	No	No	77%
	Prednisolone	Moderate inducer and substrate	Inhibitor and substrate		
Bisphophonates and Denosumab	Zoledronic acid	No	No	No	33-40%
	Pamidronic acid	No	No	No	54%
	Ibandronic acid	No	No	No	87%
	Denosumab	No	No	No	No
Antibiotics and antifungals	Fluconazol	Strong CYP3A4 inhibitor	No	No	11-12%
	Itraconazol	Strong CYP3A4 inhibitor	Inhibitor	Inhibitor	99.8%
	Clarithromycin	Strong inhibitor and substrate	Substrate	No	77%
	Levofloxacin	Moderate inhibitor and substrate	No	No	30-40%
	Doxycycline	Moderate inhibitor and substrate	No	No	82-93%
	Erythromycin	Strong inhibitor and substrate	Strong inhibitor and substrate	No	No
Antiemetic drugs	Ondansetron	Substrate	Substrate	No	70-76%
	Palonosetron	Substrate	No	No	62%
	Metoclopramide	No	No	No	No
	Aprepitant	Moderate inhibitor and substrate	No	No	97%
	Fosaprepitant	Moderate inhibitor and substrate	No	No	97%
Analgesic and anxiolytic	Oxycodone	Substrate	No	No	38-45%
	Hydromorphone	No	No	No	<10%
	Morphine	No	No	No	20-35%
	Fentanyl	Weak inhibitor and substrate	No	No	80%
	Methadone	Weak inhibitor and substrate	No	No	84-87%
	Paracetamol	Weak inhibitor and substrate	No	No	Low
	Lorazepam	No	No	No	88-92%
	Clonazepam	Substrate	No	No	86%
G-CSF	Filgrastim	No	No	No	No
ESA	Epoetin alfa/beta	No	No	No	No
	Darbepoetin alfa	No	No	No	No
Chemoprotector	Leucovorin	No	No	No	No

ESA: erythropoietin-stimulating agents; G-CSF: granulocyte colony-stimulating factor

There is some conflicting statement among the publications. When the information regarding the strength of inhibition/induction was available, it was reported in the table.

The potential mechanism of interactions described also includes weak inhibitors or inducers of the CYP3A4/P-gp [18, 19], adding more complexity to this topic. Based on the Hanigan and the TacDOAC results, it seems vital to be cautious with weak and moderate CYP3A4/P-gp inhibitors when prescribing a DOAC. Although, in these two studies were not included patients who received LMWHs, we should keep in mind that LMWHs in contrast to DOACs are not getting metabolized through CYP3A4/P-gp.

Several case reports describing DDIs between CYP3A4/P-gp inhibitors (or inducers) have been reported involving many different drug classes [20], including antivirals, anti-epileptics, antibiotics, antifungals, dexamethasone, etc., even with low doses of DOACs [21]. Therefore, there is a need to check all the drugs and not only the anticancer drugs for potential DDIs even when DOACs are utilized at a low dose.

The data on DDIs in patients with CAT are scarce. Most of the studies and cases described below included non-cancer patients with AFib or thrombosis. However, there are a few published CAT cases. Serrao A et al. [22] reported two cases of cancer patients who developed bleeding complications after receiving azacitidine and apixaban or dabigatran. Burden T et al. [23] described the case of a breast cancer patient who sustained a DDI with carbamazepine after switching from a LMWH to rivaroxaban.

It is also important to mention that DDI risk is also including other type of drugs, such as pain killer, selective serotonin reuptake inhibitors (SSRI), and antiemetics. Indeed, several of these agents are well-known substrates and/or inhibitors or CYP3A4 and/or P-gp, such as fluoxetine, paroxetine, sertraline, fentanyl, aprepitant, and fosaprepitant [13, 24].

Finally, the DDI between DOACs and other drugs involving the BCRP are still not clear. However, LMWHs do not interact with BCRP. One in vitro study confirmed that BCRP was involved in DOACs' disposition [25]. The same team reported that BCRP-mediated transport of apixaban and rivaroxaban was inhibited by 36% and 77%, respectively, in another *in vitro* study. Nevertheless, the authors also concluded that it was unlikely that a clinically significant DDIs would occur in vivo [26]. Because there are no published clinical data or case report on a potential clinically relevant DDIs involving BCRP, it is hard to make a definitive conclusion about potential DDIs with BRCP substrates, inhibitors, or inducers.

Most of the pharmacokinetic DDIs pertain the risk of CYP3A4 and/or P-gp inhibition. However, there is also a risk with CYP3A4 and/or P-gp inducers to consider.

Several studies investigated the risk of inducing the metabolism when receiving DOACs. Sennesael AL *et al.* [27] included 17 non-cancer patients mainly receiving apixaban with an inducer as a co-medication (carbamazepine, phenobarbital, phenytoin, or rifampicin). The result suggested a significant risk of reduced DOAC blood levels in patients taking strong P-gp and CYP3A4 inducers, including those without risk factors for drug accumulation. The authors reported that some actions were necessary during the follow-up for several patients, including dose increase and switch to other anticoagulants. Perlman A. et al. [28] also investigated the management of hospitalized patients with DOACs and co-administration of inducers. The authors reported that the concentration of DOACs was measured in 11 patients (10 received apixaban and one rivaroxaban) with enzyme inducers. Among apixaban-treated patients, 5 (50%) had concentrations below the 5th percentile of standard-dose apixaban in the ARISTOTLE study population. In the rivaroxaban-treated patients, the concentration measured was below the 5th percentile of rivaroxaban in the ROCKET-AF study population. It is important to notice that DOACs were used for AFib in non-cancer patients and not for CAT. Identifying this interaction led to monitoring DOACs concentration in 50% of the patients and by modifying treatment in 55% of the patients.

The drug–drug interactions between DOACs and other drugs involving human plasma proteins are still not precise. However, LMWHs do not interact with human plasma proteins.

In humans, DOACs are bound to human plasma proteins, primarily to serum albumin, the most abundant human plasma protein. The molar fractions of apixaban, edoxaban, and rivaroxaban bound to human plasma proteins are >87%, ~35%, 55%, and 95%, respectively [29]. LMWHs, in contrast, have low human plasma protein binding [30]. However, there is no study evaluating the potential impact of human plasma protein DDIs in thrombosis. Indeed, only few cases were reported over times with no clear evidence of clinical outcomes [12, 31]. As a consequence, it seems to be more a theoretical risk in thrombosis, but it is also important to consider the profile of the patients and more importantly the renal function and the nutrition status [32, 33].

## Food–Drug Interactions and Over the Counter Drugs

Food–drug interactions (FDIs) do not have the same level of concern among the medical community as DDIs. However, there are also some FDIs to consider in CAT patients. The intestinal metabolic enzyme CYP3A4 exerts its action in the proximity of P-gp in the enterocytes of the gut lumen. Simultaneous use of substrates for intestinal CYP3A4 and CYP3A4 inhibitors and inducers can change the exposure and toxicity of these drugs. For example, the area under the curve of sunitinib and nilotinib was increased by 11% and 29%, respectively, because of grapefruit, a well-known CYP3A4 inhibitor [31] (Tables 3 and 4). Some many other foods or herbs may potentially influence the pharmacokinetic parameters of oral anticoagulants. Orange, grapefruit, eucalyptus, garlic, grape juice, licorice, peppermint are the best-known herbs and foods that inhibit CYP3A4 [34]. Ginkgo biloba, berberine, green tea, grape juice, curcumin are also inhibitors of the P-gp [34]. In theory, there is a potential risk of bio-accumulation of anticoagulants substrates of CYP3A4 and/or P-gp in case of consumptions of such aliments. However, the clinical consequences are unknown

Dietary supplements are commonly used. One survey [35] reported that 89.6% of the patients had at least an occasional use of one or more dietary supplements; 78.1% reported daily use of at least one dietary supplement. The most taken dietary supplements with potential apixaban interactions and increased bleeding risk were herbal teas (11.1%) and turmeric (9%). Chinese herbs, ginger, and ginkgo biloba were used in fewer than 5% of respondents. Use of St John's wort was rare (<1%).

Although food or herbal inhibitors of CYP3A4/P-gp might interfere with the pharmacokinetics of DOACs, no direct evidence of such interactions was reported [34]. However, it highlights the complexity of the topic and the uncertainty around DDIs. Indeed, the clinical consequences of the use of a weak inhibitor of CYP3A4 and a cup of green tea and grapefruit juice in the morning was not explored.

It is also important not to focus only on potential DDIs between anticoagulants and prescribed drugs but also to consider over the counter (OTC) drugs. Tar and colleagues reported that 33% of patients had at least one OTC product with potentially serious apixaban interactions daily/most day and 6.7% took multiple products [35]. The publication did not report the clinical outcomes, but it highlighted the need to consider DDIs between DOACs and every drug, including OTC, for which the physicians have low control and may not be aware. Consequently, there is a need to routinely ask patients for any use of medications such as antiplatelets, NSAIDs.

The main challenge with food, herbs, diet supplements is the complexity of checking their usage regularly and the need to communicate updated information for each patient with different health care providers involved in the care of patients with CAT patients.

## Risk Factors for Drug–Drug Interactions (DDIs)

Several factors are associated with the development of DDIs, and most of them usually coexist in the oncological patients, including the following:

(i) Cancer and older age: DDIs are a growing concern in medicine and more specifically in cancer [36]. Leeuwen R.W. *et al.* [37] reported that 46% of cancer patients were exposed to at least one DDI. Furthermore, 14% of these DDIs were life-threatening or leading to permanent damage, and 84% of these DDIs led to the deterioration of a patient's status requiring treatment, highlighting the clinical impact of DDIs in cancer. Lechat P *et al.* [38] reported that 89.5% of elderly patients were exposed to at least one DDI. Most of these DDIs were classified as "highly clinically significant, avoid combination," or "moderately clinically significant, usually avoid combination." The latter study reported the clinical importance of DDIs in the general elderly population. In the studies mentioned above by Leeuwen and Lechat, older patients with cancer are at risk of DDIs.
(ii) Polypharmacy: is also a significant risk factor for DDIs [39]. Polypharmacy is common in cancer patients [40] and also in thrombosis [41]. Furthermore, there is a close relationship between polypharmacy and DDIs. One study [42] reported that the risk of major DDIs in cancer patients increased from 14% in patients receiving less than four medications to 24% in those receiving 4 to 7 medications and to 40% with 8 to 11 medications and finally to 67% in patients receiving more than 11 medications. The authors reported that the severity of DDIs was a significant and clinically relevant prognostic factor in advanced breast cancer [43]. Although there is a lack of data about polypharmacy in CAT patients, these patients would likely be also exposed to polypharmacy.
  Notably, one single patient might be exposed to several concomitant DDIs. In this regard, it is difficult to estimate the final effect. Indeed, the clinical impact of two moderate CYP3A4 inhibitors or two weak and one moderate CYP3A4 inhibitors in not clear. However, such might not be rare because the IPOP study reported that among 924 elderly non-cancer patients, 77.0% of them were exposed to at least two DDIs [38].

(iii) Patient's profile regarding comorbidities and organs' dysfunction: is also of great relevance. One pharmacokinetic study reported increased area under the curve of rivaroxaban in patients with mild renal impairment exposed to verapamil [44]. Patients with cancer are at increased risk of renal impairment, and around 50% of cancer patients are exposed to renal insufficiency [45]. Mild renal impairment is not enough to induce bioaccumulation of rivaroxaban, but this study highlights that the combination of several bioaccumulative risk factors could influence the pharmacokinetics of a drug. Gong IY *et al.* [46] also reported that moderate hepatic impairment, renal impairment, ethnicity, weight, and age could influence the pharmacokinetic of DOACs.
(iv) Pharmacological characteristics of the drugs: have a significant impact on the risk and characteristics of the DDIs. In general, a lower risk is associated with monoclonal antibodies and a higher risk with chemical agents such as cytotoxic chemotherapies or tyrosine kinase inhibitors. However, there are some exceptions, and monoclonal antibodies could also interact with CYP3A4. For example, brentuximab is a CYP3A4 substrate and a potential inhibitor of CYP3A4 [13, 47] (Tables 3 and 4). Brentuximab is an antibody-drug conjugate composed of the anti-CD30 chimeric monoclonal antibody and the potent antimicrotubule drug monomethylauristatin E (MMAE). This MMAE is a substrate of CYP3A4 and not the monoclonal antibody itself (SmPC, Adcetris®, EMA). Hence, it is essential to check for potential DDIs with the main compounds and the metabolites or "side" compounds and not automatically exclude a risk of DDIs because of the nature of a drug.

## Guidelines, Algorithms, and Recommendations

The guidelines from international scientific societies highlight the importance of potential DDIs when choosing an anticoagulant [3–5] in CAT patients. Several publications follow an algorithm approach.

The American Society of Clinical Oncology (ASCO) [5] describes that one important safety consideration in using any DOACs in CAT patients is the potential for DDIs. Potent inhibitors/inducers of P-gp and potent inhibitors or inducers of CYP3A4 can interact with DOACs. The American Society of Hematology (ASH) states that the choice of anticoagulant treatment must be based on the specific clinical setting, including DDIs [4]. International Initiative on Thrombosis and Cancer (ITAC) also indicates that DOACs pose challenges in oral administration and DDIs [3].

Carrier M. et al. published the first CAT treatment algorithm [48]. This algorithm describes how to manage CAT and, more specifically, how to choose between LMWHs and DOACs. According to the treatment algorithm, patients considered to be at high risk of bleeding, those with active gastrointestinal or urothelial cancer, or those taking concomitant medications that would lead to potentially serious DDIs with DOACs should be treated with a therapeutic dose of extended-duration LMWHs. Later, two other algorithms [49, 50] also had very similar recommendations.

Terrier J. et al. [51] published a practical recommendation regarding the use of anticoagulants when facing potential DDIs, in non-cancer patients. Interestingly, in the case of moderate/weak P-gp inhibitor only, the authors recommend using DOACs with caution and weight benefit-risk ratio or consider VKAs as a first-line treatment if patients possess at least two risk factors. LMWHs were not mentioned, likely because Terrier J. et al. focused on non-cancer patients. The same recommendation was made in moderate/weak CYP3A4/5 inhibitor only for apixaban and rivaroxaban. Finally, in the case of CYP3A4/5 or combined Pgp/CYP3A4/5 inducer, co-administration is not recommended.

The indication of VKAs is also questionable. Indeed, guidelines are still giving some space for VKAs. The ASH CAT guidelines suggested to use low-dose acetylsalicylic acid or fixed low-dose VKA or LMWH for multiple myeloma patients receiving lenalidomide, thalidomide, or pomalidomide-based regimens [4]. The ASCO guidelines are also considering VKAs for the long-term anticoagulation if LMWHs or DOACs are not accessible. The same guideline is also stating that anticoagulation with LMWHs, DOACs, or VKAs beyond the initial 6 months should be offered to select patients with active cancer [5]. Finally, besides the fact that VKAs need a regular monitoring using INR, it could may be a potential solution when adherence is challenging.

## Conclusion

DDIs are associated with an increased risk of bleeding or recurrent VTE in patients with CAT. Moreover, the DDIs may reduce the efficacy and safety of anticancer therapies or other drugs used for concomitant medical conditions. The clinical evidence on the impact of DDIs in daily practice is minimal, particularly in patients with cancer.

The recent RCTs designed to compare the efficacy and safety of DOACs vs LMWHs in patients with CAT excluded those at risk of potential DDIs. Consequently, the available data are minimal and particular attention must be taken into account when prescribing anticoagulants in this setting. Further prospective research on this topic would be challenging from a practical and ethical point of view since it would expose patients to either bleeding risk (with inhibitors) or VTE recurrence (with inducers). Moreover, the DDIs could lead to a decrease in the efficacy of antineoplastic therapies. Consequently, the assessment of potential DDIs in patients with CAT is currently based on the empirical estimation according to the metabolic profile of each drug and data from case reports and retrospective studies. Thus, it is advisable to reassess periodically on changes in medications and the potential DDIs. The development of programs aimed at promoting the education and participation of patients to report changes in medications and eating habits or the use of herbal supplements would be of interest in this setting. Moreover, the implementation of automatic systems to cross data from the pharmacy and the clinicians involved in the care of patients in order to activate alerts for possible DDIs would be helpful.

However, several studies described earlier are a good start to understanding the concern about DDIs in CAT with an increased risk of bleeding with inhibitors and an increased risk of VTE with inducers. The risks are higher with DOACs, while LMWHs are not linked to pharmacokinetic DDIs.

It is also essential to have a global and pragmatic approach when managing DDIs. Several factors should be considered, such as age, comorbidities, polypharmacy, the severity of cancer. It is also important to check for DDIs when a new drug is introduced. This may be due to the change in cancer treatment or other cancer or non-cancer-related situations. A patient may face an infection during his treatment journey, and Hill [15] reported that antibiotics could negatively impact the outcomes in DDIs with DOAC. Therefore, it is crucial to consider the regular review of potential DDIs because the patient profile, cancer, comorbidities, and all the treatments evolve over time, highlighting the dynamic aspects of the diseases.

In conclusion, DDIs are a challenge in cancer patients and patients with CAT. More attention should be given to the recognition of potential drug-drug interactions in the initial anticoagulation management as well as in the anticancer treatment. Regular assessment of potential drug–drug interaction should be implemented, and therapies should be adapted according to the risk and the patients' needs. Last but not least, we should keep in mind that drugs are treating a patient and not a disease, meaning that there is value when implementing a personalized approach. Inclusion of pharmacist on the treatment and oversight team and automated electronic drug interaction analysis is vital.

## Data Availability

Not applicable for this article

## Code availability

Not applicable for this article
