# Correction to: Complexity and clinical significance of drug–drug interactions (DDIs) in oncology: challenging issues in the care of patients regarding cancer‑associated thrombosis (CAT)

**DOI:** 10.1007/s00520-022-07329-3

**Published:** 2022-08-25

**Correction to: Supportive Care in Cancer**

**https://doi.org/10.1007/s00520-022-07235-8**

The online version of the original paper contained errors due to an updated file released but the pdf is correct. The purpose of this Erratum is the correction of the online version in the indexing sites.

The online version of the original article has been corrected

